# Supplementary material for: Placental cell type deconvolution reveals that cell proportions drive preeclampsia gene expression differences
Source: Commun Biol. 2023 Mar 13;6:264. doi: 10.1038/s42003-023-04623-6 (PMC10011423; doi:10.1038/s42003-023-04623-6)
Supplement: Supplementary file 2 — Supplementary Information [file 42003_2023_4623_MOESM2_ESM.pdf]

Title: Placental cell type deconvolution reveals that cell proportions drive preeclampsia gene expression differences

Authors: Campbell KA[1], Colacino JA[2,3], Puttabyatappa M[4], Dou J[1], Elkin ER[2], Hammoud SS[5,6], Domino SE[6], Dolinoy DC[2, 3], Goodrich JM[2], Loch-Caruso R[2], Padmanabhan V[2,3,4,6], Bakulski KM[1]*

Affiliations:

[1]Epidemiology, School of Public Health, University of Michigan

[2]Environmental Health Sciences, School of Public Health, University of Michigan

[3]Nutritional Sciences, School of Public Health, University of Michigan

[4]Pediatrics, Michigan Medicine, University of Michigan

[5]Human Genetics, Michigan Medicine, University of Michigan

[6]Obstetrics and Gynecology, Michigan Medicine, University of Michigan

[7]Department of Urology, Michigan Medicine, University of Michigan

Supplementary Figures and Tables.

This document contains 15 Supplementary Figures and 3 Supplementary Tables.

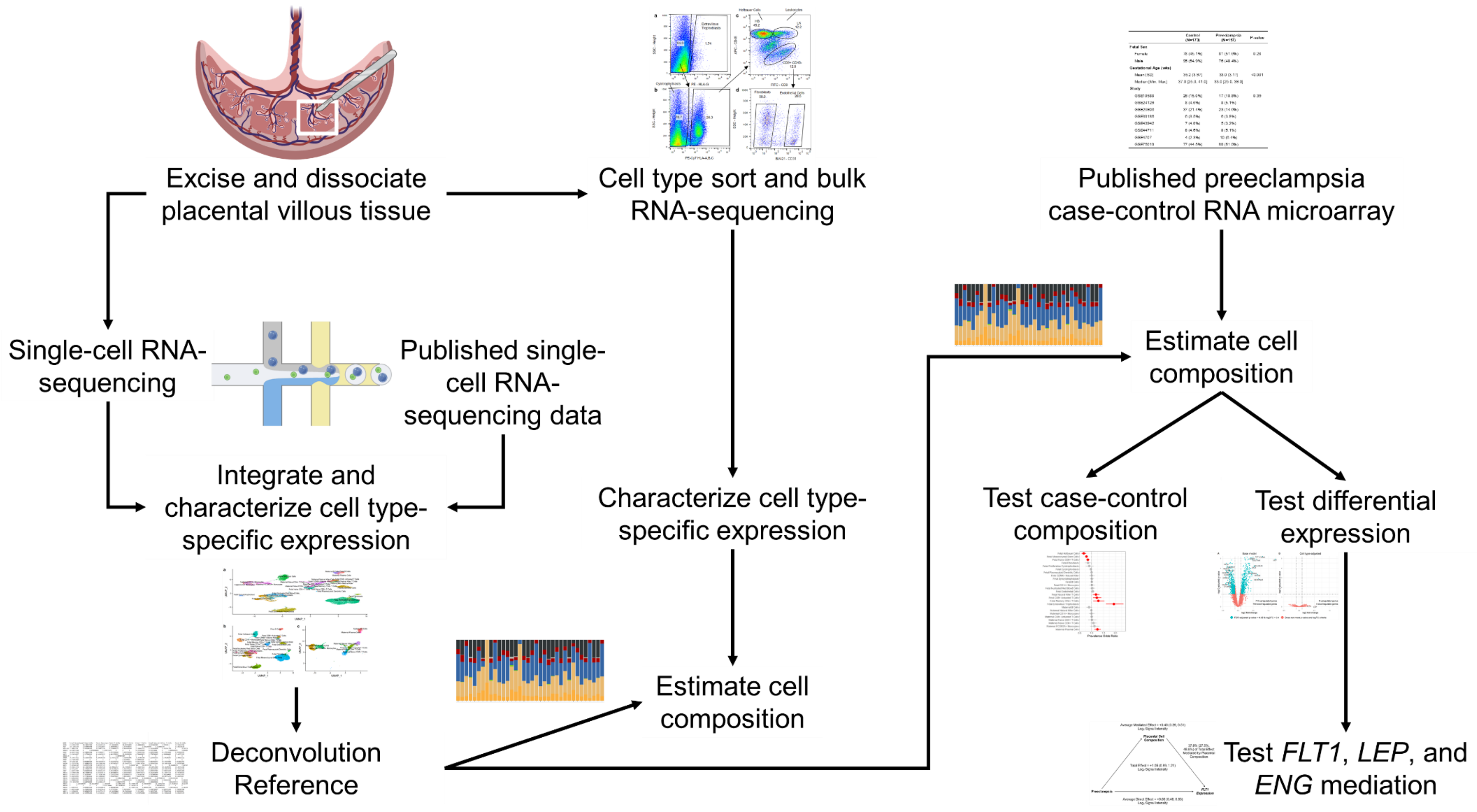

Supplementary Figure 1. Conceptual layout of the laboratory methods and analyses contained within this manuscript. Created with BioRender.com.

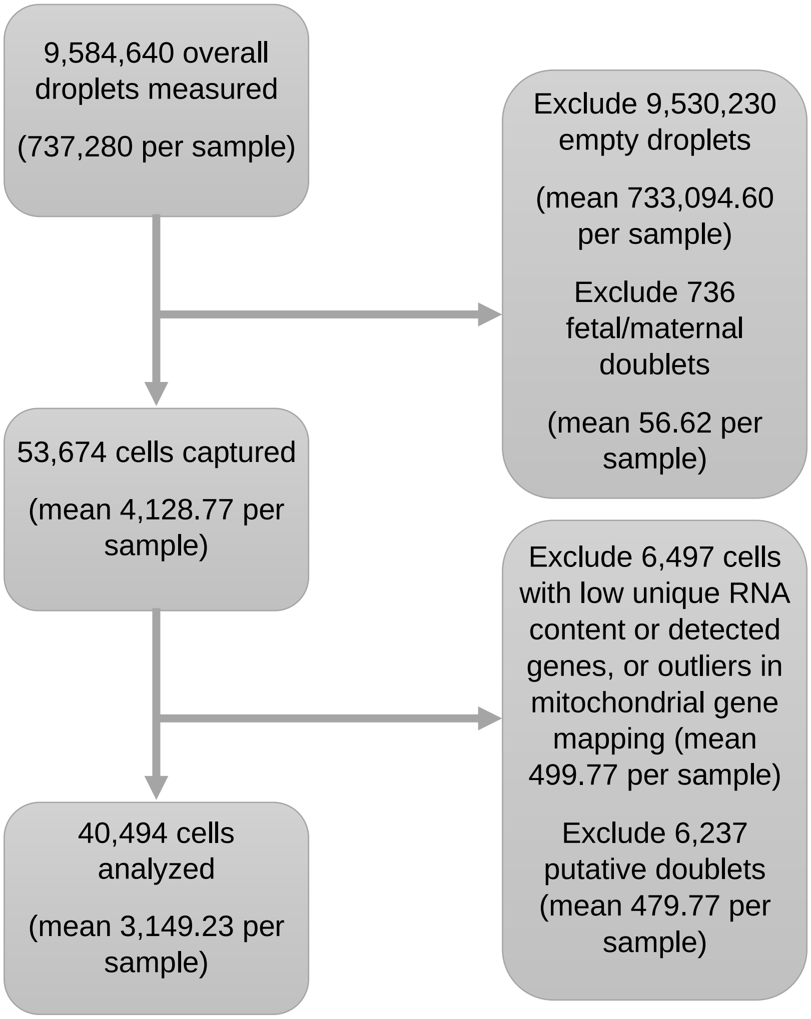

Supplementary Figure 2. Placental single-cell RNA sequencing quality control pipeline

Supplementary Table 1. Summary of single-cell RNA-sequencing sample characteristics and sequencing quality metrics. This study collected Samples 1-2. Samples 3-5 were downloaded from Pique-Regi et al., 2019 [1]. Samples 6-9P were download from Tsang et al., 2017 [2]. For Samples 1-2, A/B pairs are technical replicates. For Samples 8-9, C/P pairs represent centrally (C) or peripherally (P) sampled villous tissue from the same placenta.

		Pre-filtering Quality Control Metrics					Post-filtering Quality Control Metrics							
Sample	Fetal Sex	Droplets Sequenced	Total Unique RNA Molecules	Total Unique Genes Detected	Total Cells	Maternal-Fetal Doublets Removed	Unique RNA Molecules (Median)	Unique RNA Molecules (IQR)	Unique Genes (Median)	Unique Genes (IQR)	Percent Mitochondria Gene Expression (Median)	Percent Mitochondria Gene Expression (IQR)	Putative Doublets Removed	Cells in Final Analytic Sample
1A	F	737,280	15,329,288	32,738	2,573	28	4,021	2,717	1,247	442	4.1	1.98	116	2,214
1B	F	737,280	14,777,010	32,738	2,600	33	3,870	2,521	1,189	426	4.06	1.85	134	2,280
2A	M	737,280	14,306,604	32,738	2,544	25	3,988	2,448	1,171	430	2.86	1.48	92	2,292
2B	M	737,280	14,799,594	32,738	2,740	29	3,875	2,410	1,157	427	2.85	1.56	109	2,458
3	M	737,280	17,075,126	36,601	1,907	0	3,556	6,830	1,292	1,781	2.76	2.89	105	1,620
4	F	737,280	28,250,436	36,601	2,653	4	5,352	12,664	1,833	2,869	3.73	3.79	119	2,081
5	M	737,280	29,693,207	36,601	2,456	1	6,544	10,889	2,186	2,569	2.25	1.94	136	2,210
6	M	737,280	71,470,103	36,601	6,018	401	1,639	1,996	733	716	3.02	4.2	619	3,765
7	M	737,280	18,472,614	36,601	16,968	206	1,617	3,237	687	844	2.66	2.91	3,860	10,679
8C	F	737,280	32,463,561	36,601	4,918	5	1,880	1,986	793	631	3.09	3.72	533	3,726
8P	F	737,280	62,317,586	36,601	2,284	3	1,302	1,608	585	567	3.35	4.24	219	1,651
9C	F	737,280	20,847,761	36,601	3,137	0	2,173	2,242	875.5	690	1.92	2.31	247	2,508
9P	F	737,280	64,935,823	36,601	3,612	1	2,073.5	2,088	879	648	2.06	2.97	208	3,010

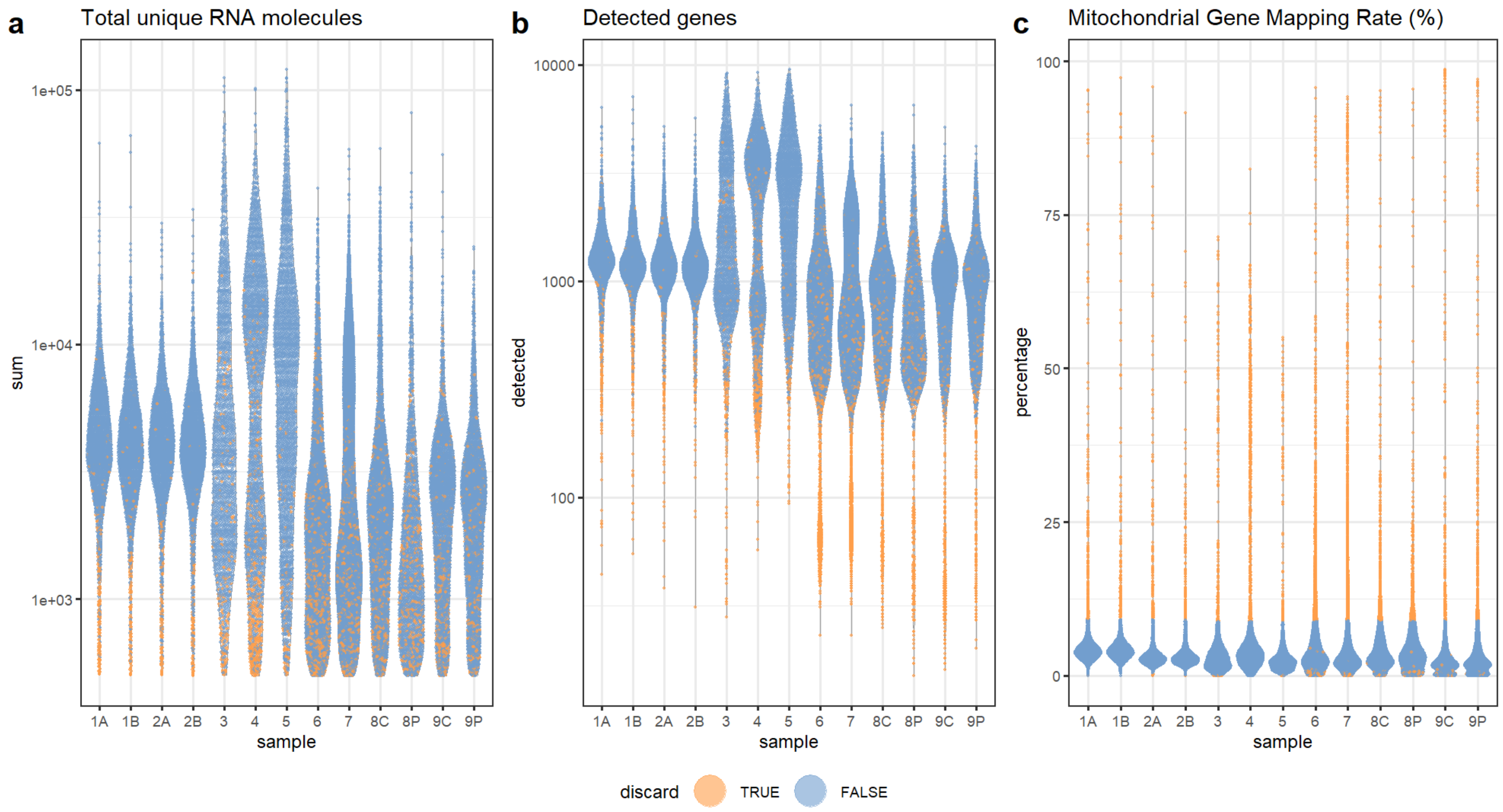

Supplementary Figure 3. Placental single cell RNA sequencing of quality metrics by sample, visualized using violin plots. Orange cells were discarded based on outlier status on any of the following metrics: (a) total unique RNA transcripts (also called unique molecular identifiers) < 500, (b) number of genes expressed < 200, or (c) outliers in percent mitochondrial genes expressed.

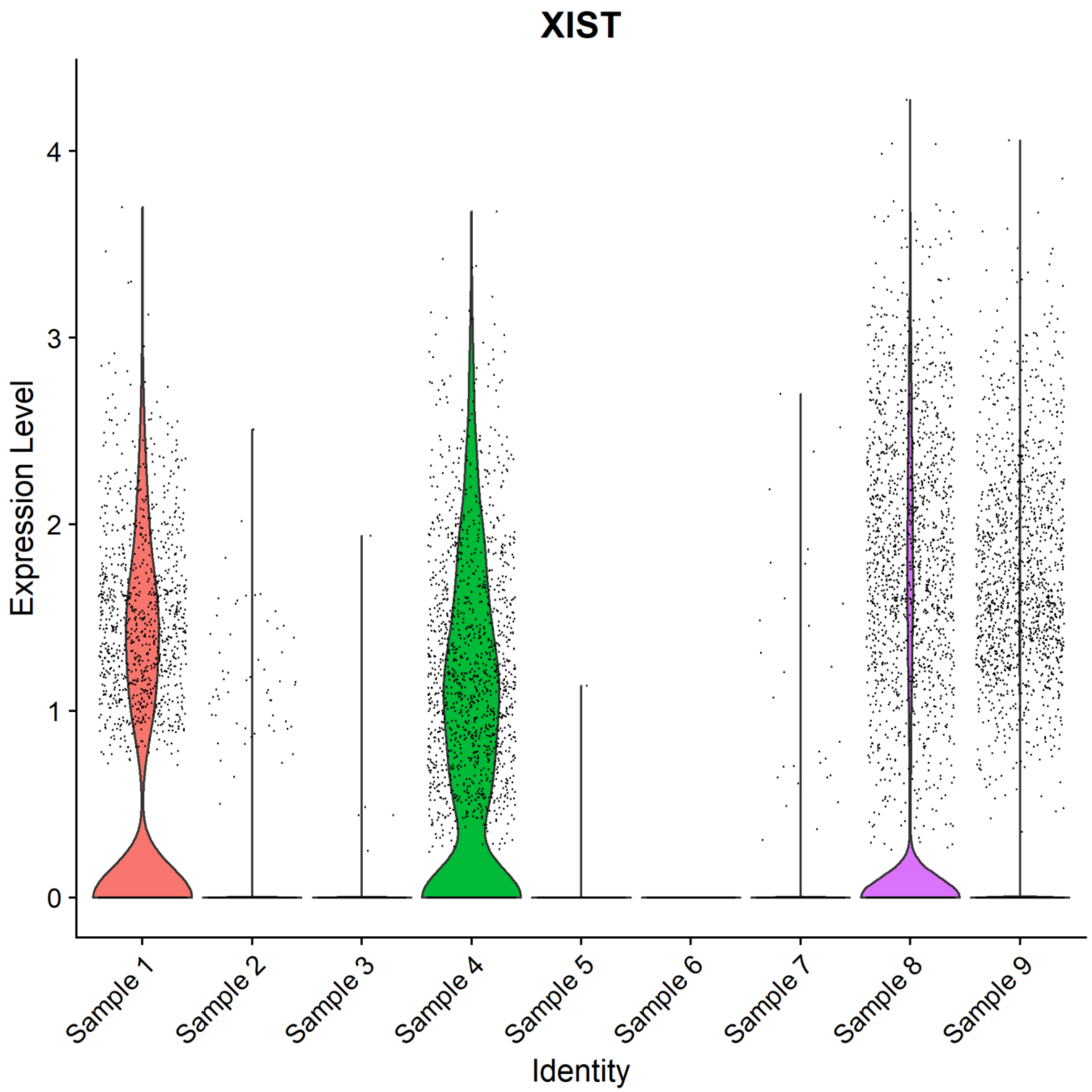

Supplementary Figure 4. Library size-normalized and log-transformed *XIST* expression in fetal origin cells by biological replicates identifies Sample 1 as female due to high *XIST* expression, Sample 2 as male, and confirms fetal sex annotation for Samples 3-9.

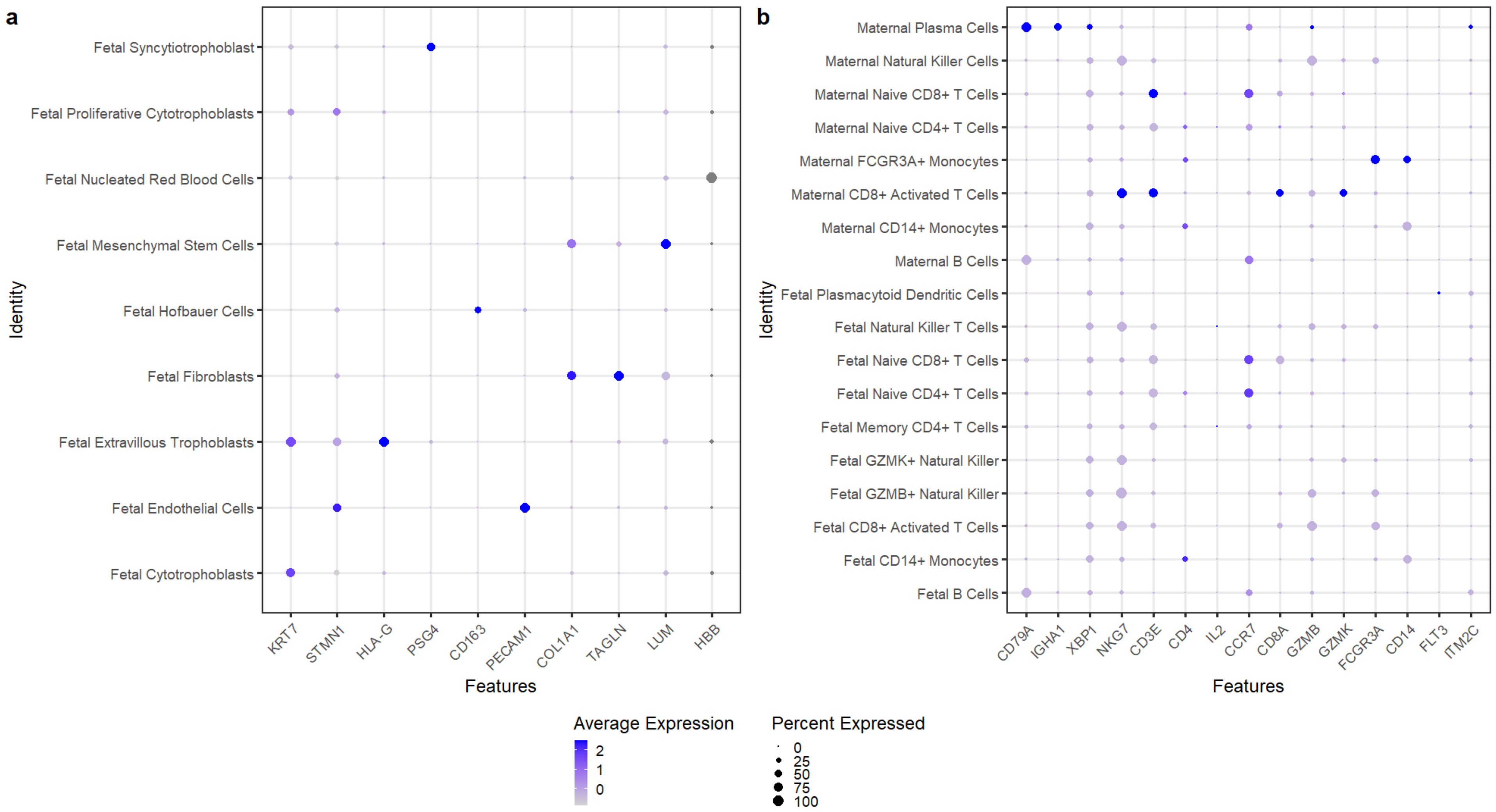

Supplementary Figure 5 Dot plots of cell type marker genes used to annotate cell clusters to cell describing average normalized gene expression types (color darkness) and percentage of cells in a cell type cluster expressing that gene (point size). (a) Placental tissue cell types (b) Peripheral blood cell types

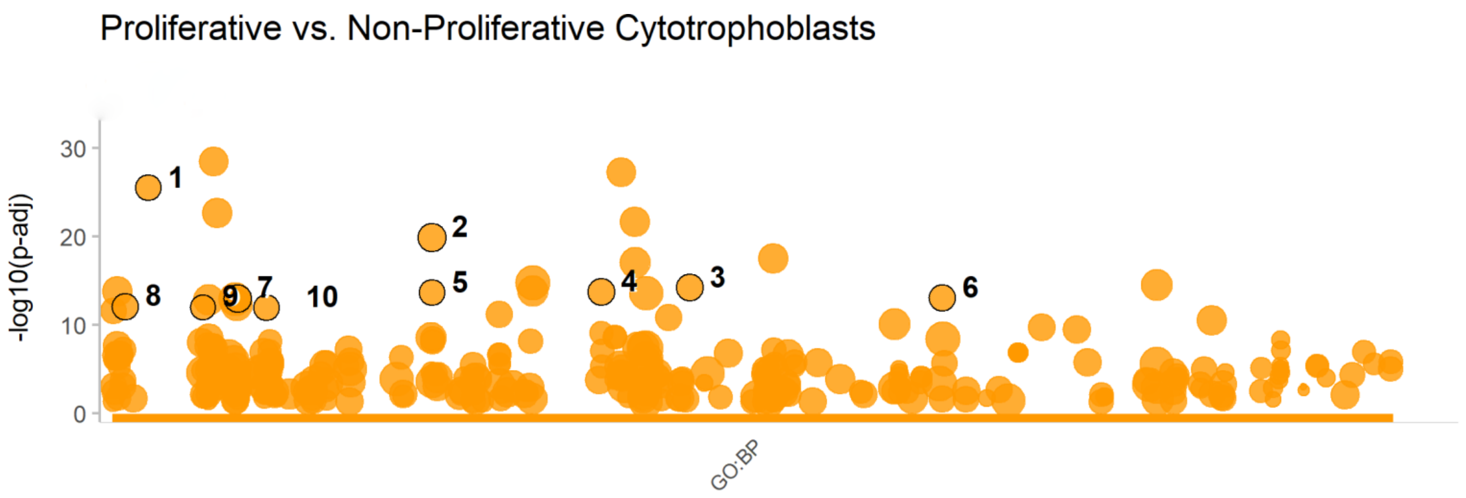

id	source	term_id	term_name	term_size		p_value
1	GO:BP	GO:0002181	cytoplasmic translation	144		3.4e-26
2	GO:BP	GO:0022613	ribonucleoprotein complex biogenesis	353		1.3e-20
3	GO:BP	GO:0046034	ATP metabolic process	226		6.5e-15
4	GO:BP	GO:0042254	ribosome biogenesis	241		1.9e-14
5	GO:BP	GO:0022618	ribonucleoprotein complex assembly	163		2.4e-14
6	GO:BP	GO:0071826	ribonucleoprotein complex subunit organization	170		9.3e-14
7	GO:BP	GO:0007059	chromosome segregation	301		1.0e-13
8	GO:BP	GO:0000819	sister chromatid segregation	190		8.4e-13
9	GO:BP	GO:0006119	oxidative phosphorylation	120		9.9e-13
10	GO:BP	GO:0009060	aerobic respiration	154		1.1e-12

g:Profiler (biit.cs.ut.ee/gprofiler)

Supplementary Figure 6. Top biological process gene ontology enrichment results with between 15 and 500 annotated genes for proliferative vs. non-proliferative cytotrophoblasts overexpressed differential expression results.

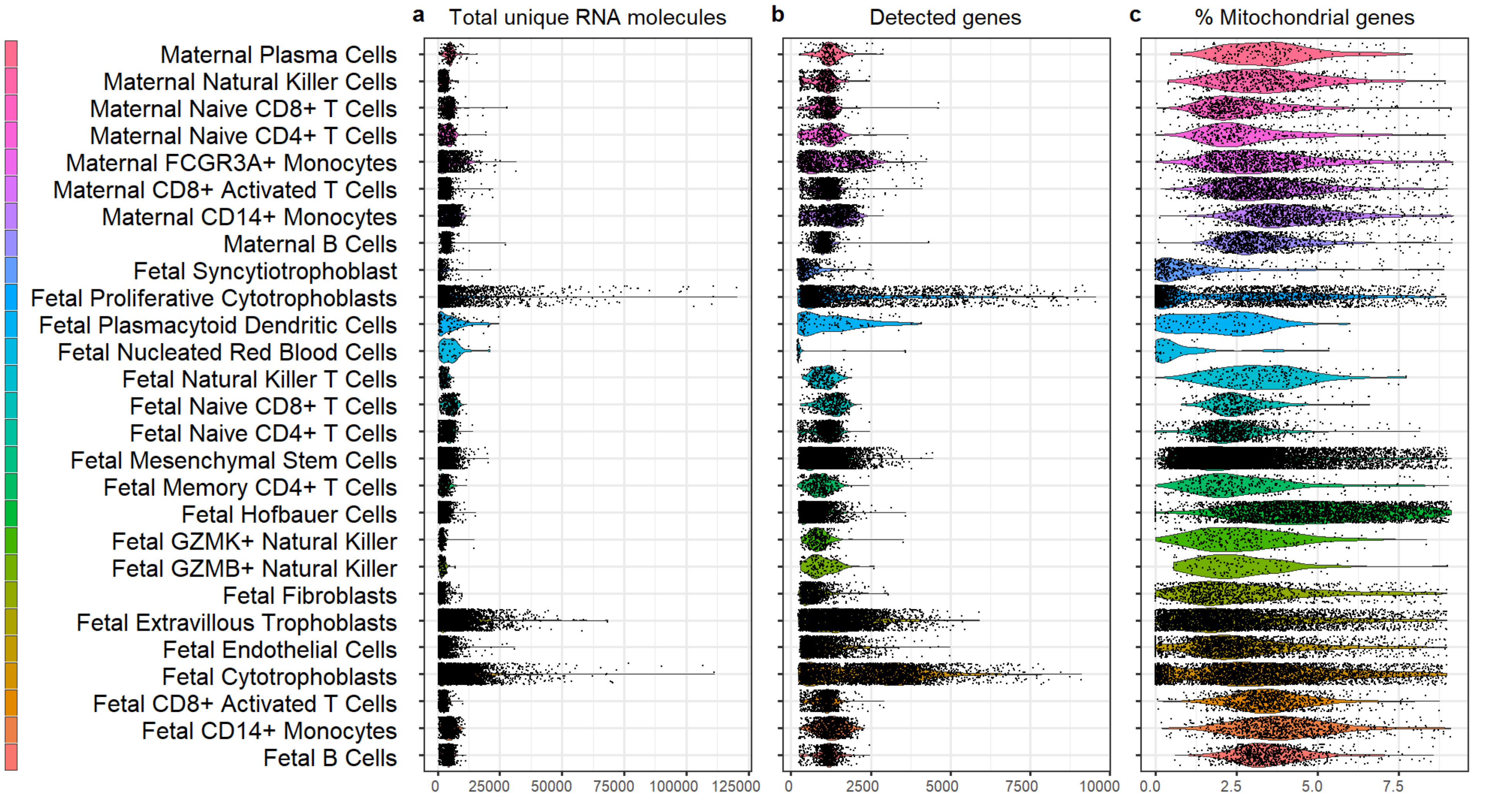

Supplementary Figure 7. Placental single cell RNA sequencing of quality metrics by cluster, visualized using violin plots. (a) Number of genes expressed, (b) total unique RNA molecules, and (c) percent mitochondrial genes expressed.

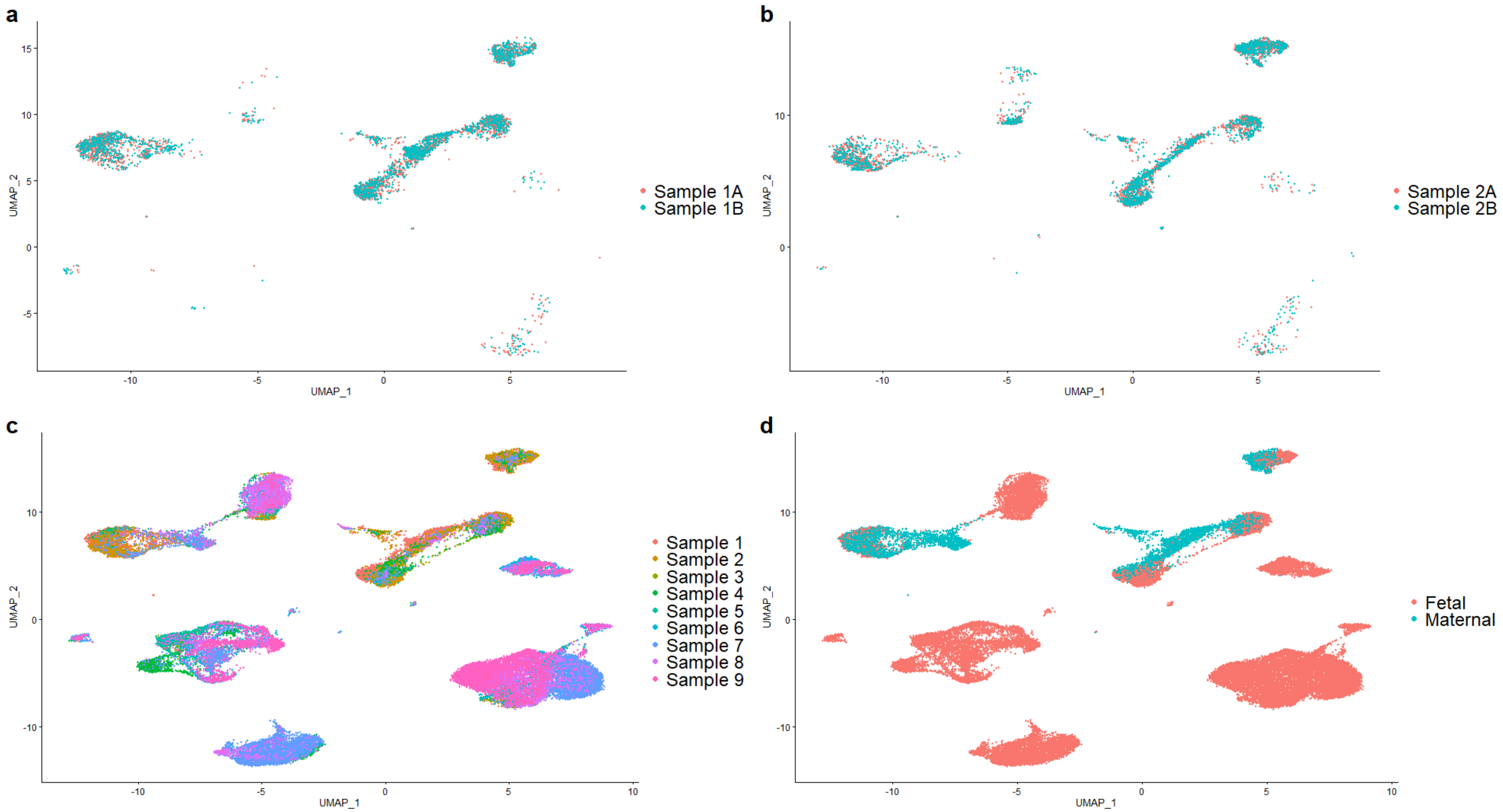

Supplementary Figure 8. Uniform Manifold Approximation and Projection (UMAP) plots colored by key variables. (a) Technical replication in Sample 1 with points colored by technical replicate. (b) Technical replication in Sample 2 with points colored by technical replicate. (c) Biological replicates identified by point color with collapsed technical replicates. (d) Fetal/Maternal origin assignment by point color.

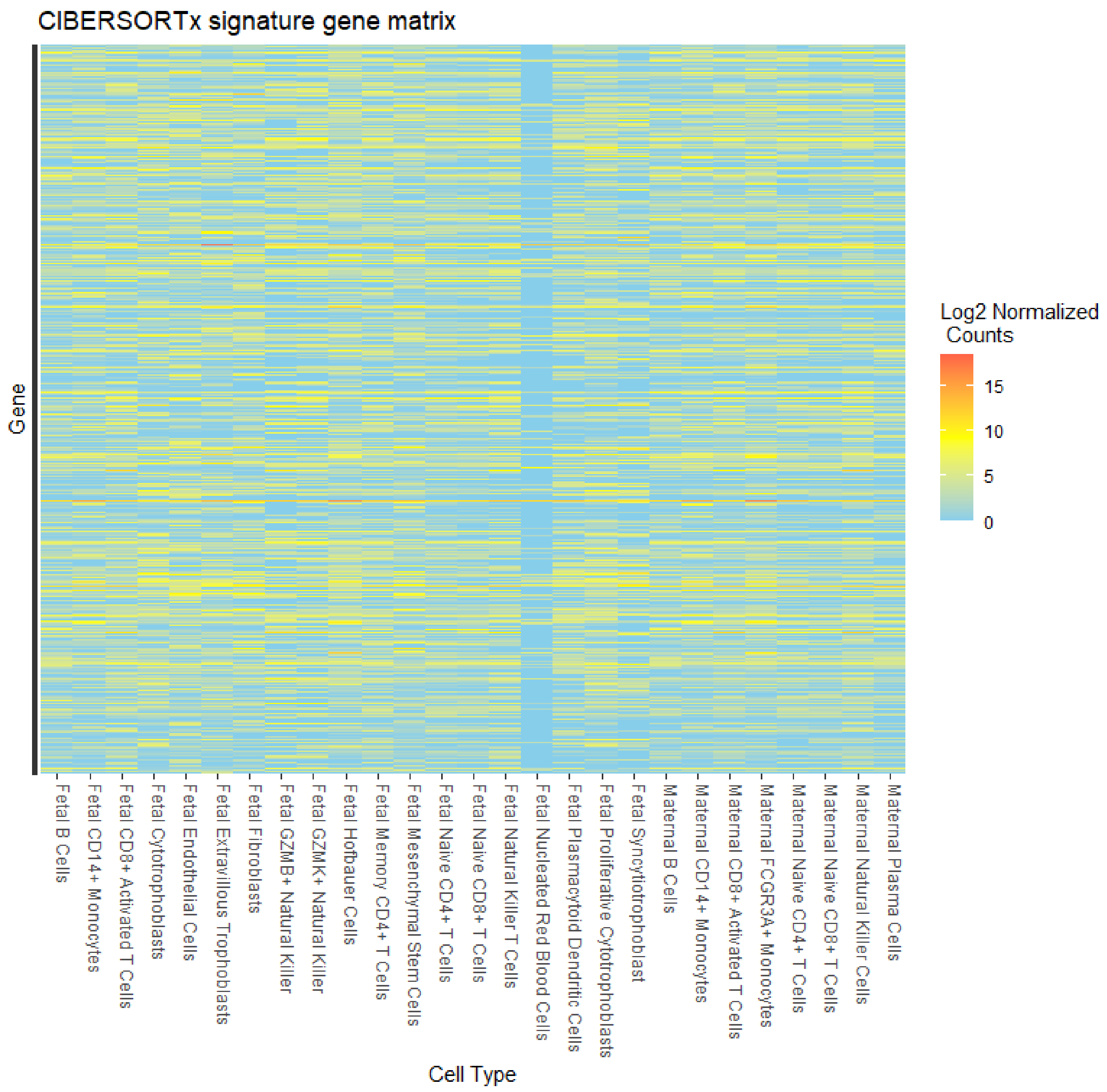

Supplementary Figure 9. Heatmap of signature gene expression matrix in log_2-transformed library size-normalized counts (counts per million) generated and used to deconvolute bulk placental tissue dataset. Cell types are encoded on the y-axis and genes are located along the x-axis. Blue indicates low expression of a gene and red represents high expression.

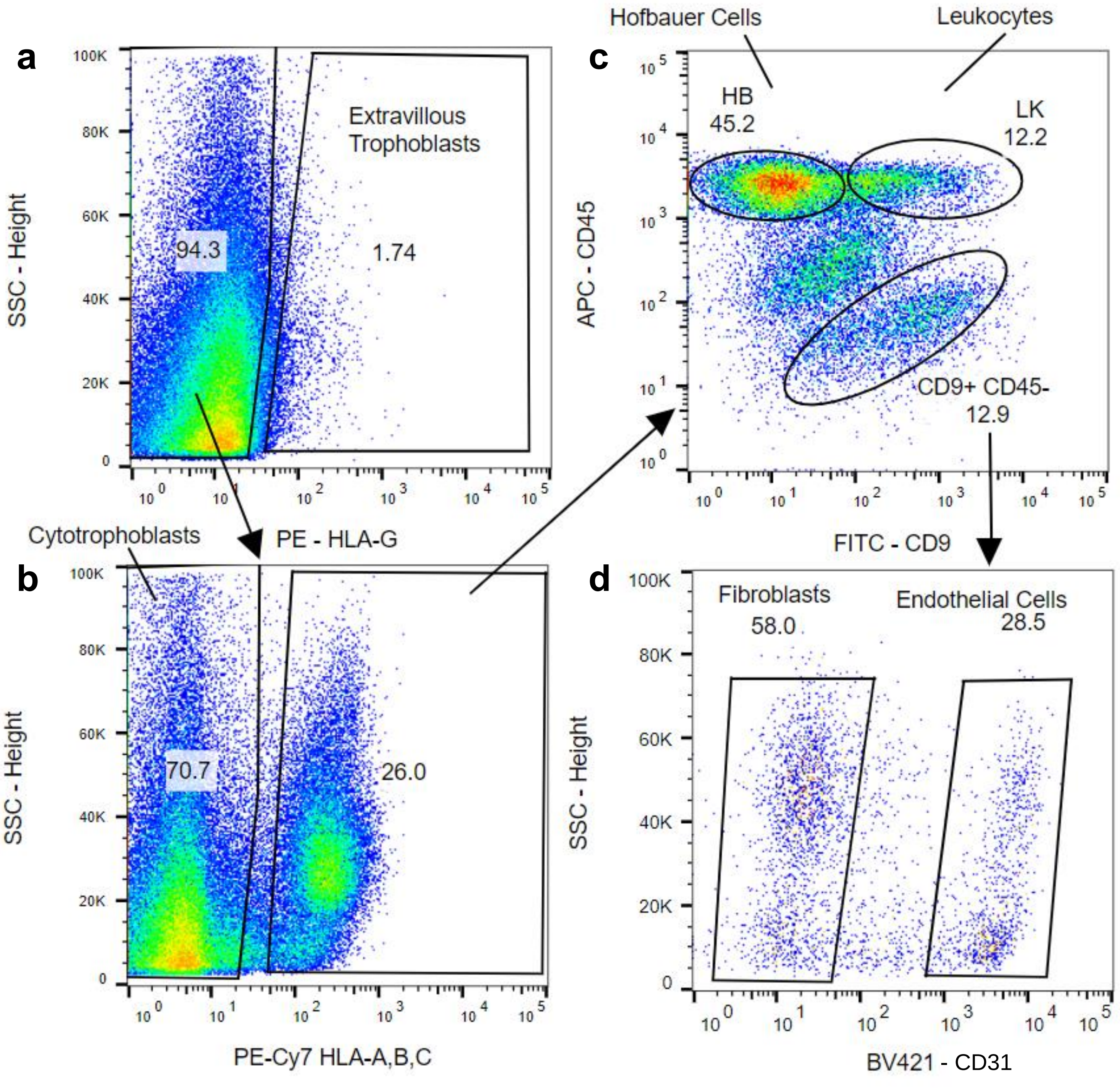

Supplementary Figure 10. A representative FACS sort. Gating strategy: (a) HLA-G/PE to positively select extravillous trophoblasts; (b) HLA-ABC/PE-CY7 to negatively enrich for cytotrophoblasts; (c) CD9/FITC by CD45/APC to positively select for Hofbauer cells and leukocytes; (d) CD31/BV421 to distinguish endothelial cells from CD31- fibroblasts.

Supplementary Table 2. Fluorescence-activated cell sorting and RNA-sequencing quality control results. Each sample was sorted into six cell type populations with a matched whole tissue sample. Cell count describes the total number of FACS-sorted cells. Table columns describe total RNA given in nanograms with RNA integrity index score (RIN), fastQC pass/fail, and whether the sample was sequenced in the paired-end (PE) or single-end (SE) and included in the experiment based on RIN score and total RNA (all sequenced samples included).
*Matched with sample 1 from the single-cell RNA-sequencing assay.

Sample ID	Cell Type	Cell Count	Total RNA (ng)	RIN	fastQC	Sequenced (PE/SE)
Sorted 1*	Syncytiotrophoblast	N/A	0.2	1	N/A	Dropped
	Hofbauer	2.00E+05	4.2	5	Pass	SE
	Leukocyte	1.57E+05	6.9	7.3	Pass	SE
	Extravillous Trophoblast	8.35E+03	1.1	6.4	Pass	SE
	Cytotrophoblast	2.83E+05	1.8	8.1	Pass	SE
	Fibroblast	1.55E+04	34	7.3	Pass	SE
	Endothelial Cells	7.60E+03	3	2.8	N/A	Dropped
	Whole Tissue	N/A	108	4.9	Pass	SE
	Whole Tissue	N/A	209	7.8	Pass	PE
Sorted 2	Syncytiotrophoblast	N/A	0.2	1	N/A	Dropped
	Hofbauer	1.57E+05	1.6	4.9	Pass	SE
	Leukocyte	1.37E+05	63	8.3	Pass	SE
	Extravillous Trophoblast	3.60E+03	1.1	6	Pass	SE
	Cytotrophoblast	8.00E+04	<0.1	<0.1	N/A	Dropped
	Fibroblast	1.06E+04	35	8.1	Pass	SE
	Endothelial Cells	2.30E+03	2	8.1	Pass	SE
	Whole Tissue	N/A	332	8.7	Pass	SE
Sorted 3	Syncytiotrophoblast	N/A	255	4	Pass	SE
	Hofbauer	1.02E+05	45	9.1	Pass	SE
	Leukocyte	8.30E+04	50	8.5	Pass	SE
	Extravillous Trophoblast	2.50E+04	7.2	2.8	Pass	SE
	Cytotrophoblast	3.72E+05	3.7	2.4	N/A	Dropped
	Fibroblast	3.61E+03	2.9	1.3	N/A	Dropped
	Endothelial Cells	2.97E+03	3	2.5	N/A	Dropped
	Whole Tissue	N/A	9572	5.3	Pass	SE
Sorted 4	Syncytiotrophoblast	N/A	107	6.5	Pass	PE
	Hofbauer	8.40E+04	87	6.7	Pass	PE
	Leukocyte	2.20E+04	0.82	7.6	Pass	PE
	Extravillous Trophoblast	4.50E+04	2.4	8	Pass	PE
	Cytotrophoblast	2.56E+05	1.5	6.9	Pass	PE
	Fibroblast	1.70E+04	1.7	7.2	Pass	PE
	Endothelial Cells	1.20E+04	0.14	1	Pass	Dropped
	Whole Tissue	N/A	298	8.2	Pass	PE

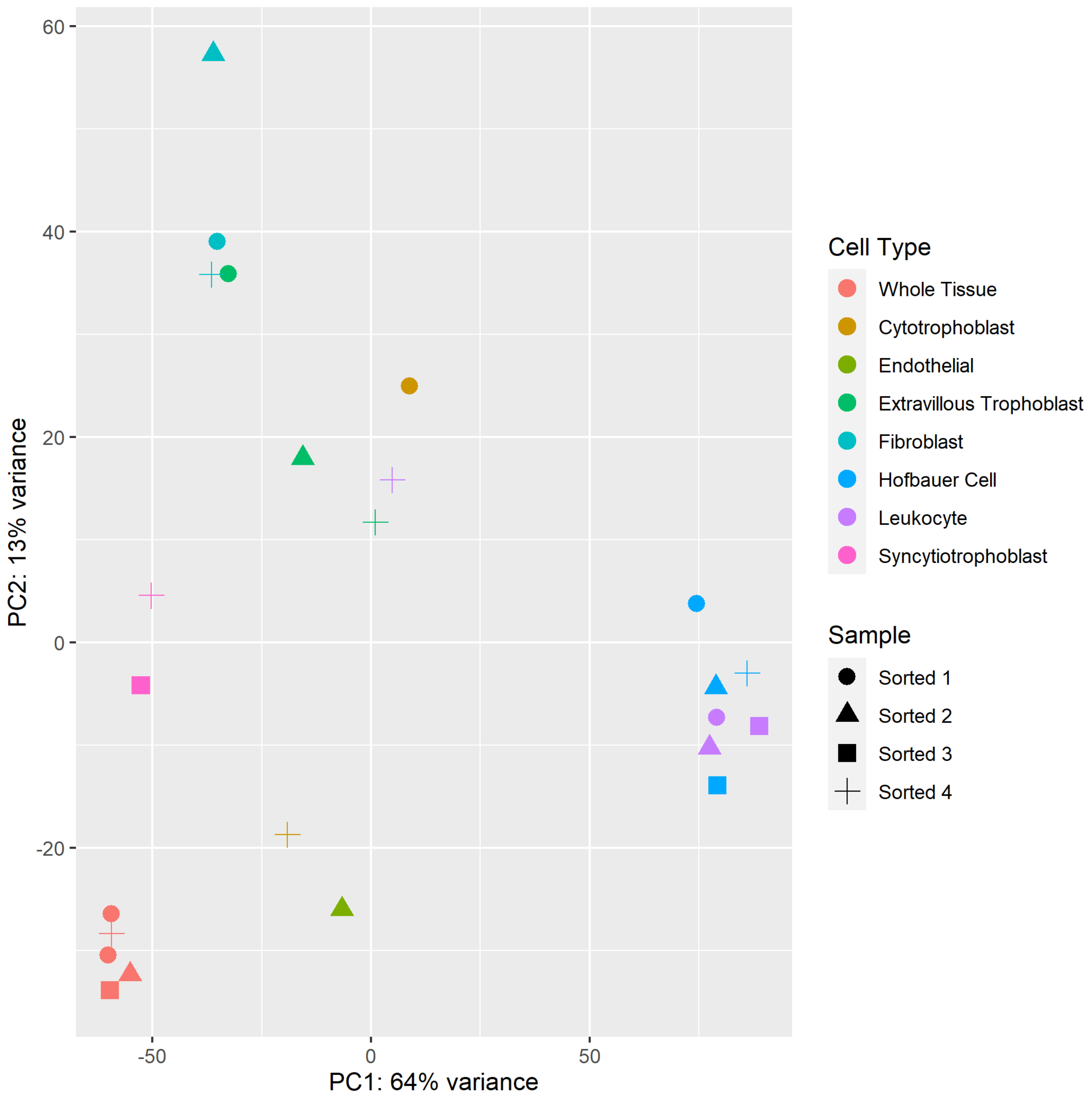

Supplementary Figure 11. Principal components plot of fluorescence-activated cell sorting bulk RNA-sequencing results on sorted placenta samples. Point colors encode cell type. Shape denotes sample source.

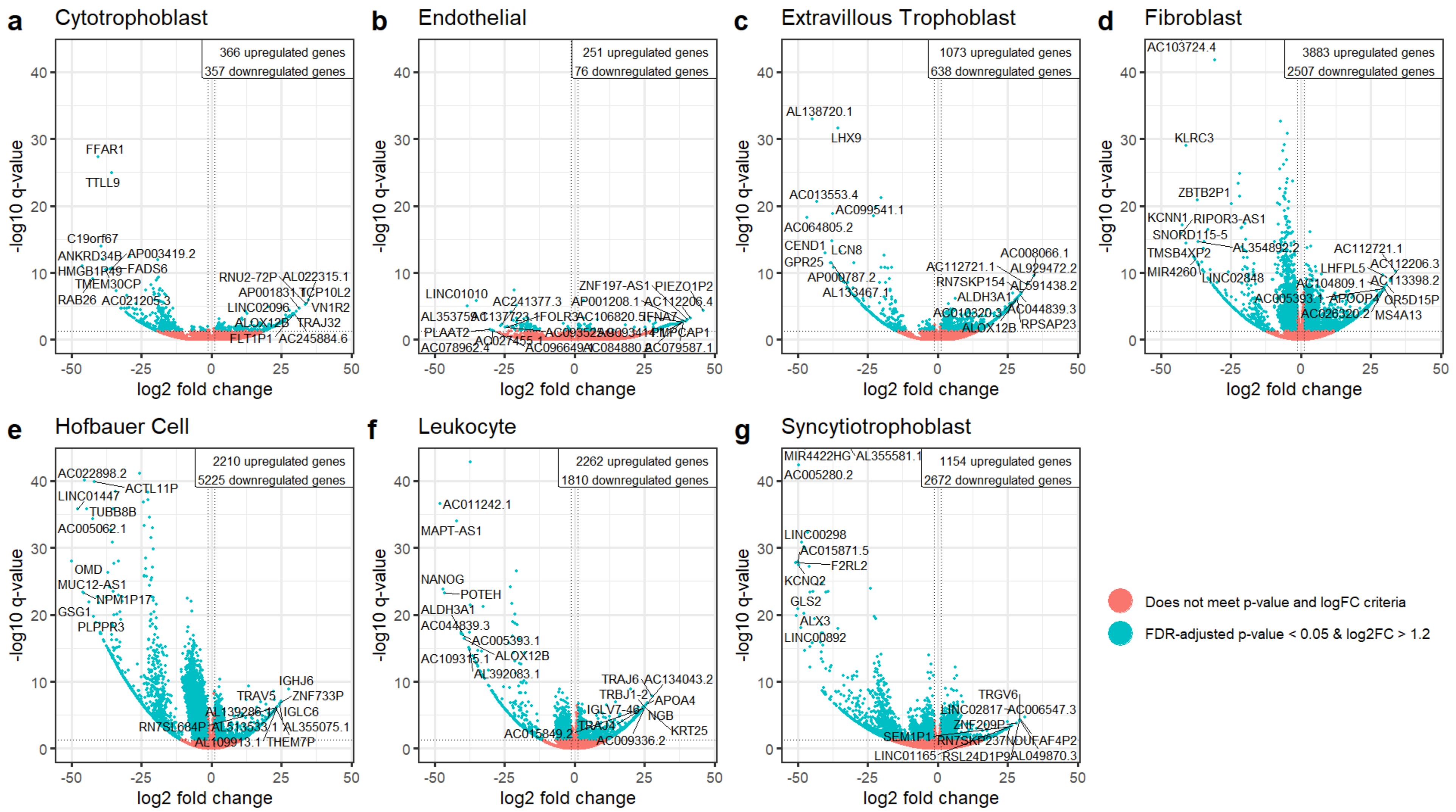

Supplementary Figure 12. Volcano plots for fluorescence-activated-cell-sorted bulk RNA-seq differential expression in one cell type against average gene expression across other cell types. The y-axis encodes -log10 transformation of the false discovery-controlled q-value, with the cut-off for statistical significance at 0.05. The x-axis encodes log2 fold change of gene expression for the contrast of interest. The upper-right inset describes the number of differentially regulated genes per contrast. 37,929 genes were tested. 746 genes were dropped from the syncytiotrophoblast contrast by DESeq2's default automatic filtering algorithm due to excessively low counts, low variability, or extreme outlier status. (a) Cytotrophoblast. (b) Endothelial cell. (c) Extravillous trophoblast. (d) Fibroblast. (e) Hofbauer cell. (f) Leukocyte. (g) Syncytiotrophoblast.

Cell Type	Single-cell Differentially Expressed Genes	Sorted Differentially Expressed Genes	Overlapping Genes	Percentage overlap (single-cell denominator)	Percentage overlap (sorted denominator)
Cytotrophoblast*	2011	366	13	0.6%	3.6%
Endothelial Cells	769	251	1	0.1%	0.4%
Extravillous Trophoblasts	693	1073	18	2.6%	1.7%
Fibroblasts**	649	3883	310	47.8%	8.0%
Hofbauer Cells	297	2210	77	25.9%	3.5%
Leukocytes***	3378	2262	486	14.4%	21.5%
Syncytiotrophoblast	206	1154	28	13.6%	2.4%
Mean:	1,143	1,600	133	15.0%	5.9%
Cell Type	Single-cell Overrepresented Pathways	Sorted Overrepresented Pathways	Overlapping Pathways	Percentage overlap (single-cell denominator)	Percentage overlap (sorted denominator)
Cytotrophoblast*	98	32	12	12.2%	37.5%
Endothelial Cells	243	6	0	0.0%	0.0%
Extravillous Trophoblasts	59	18	8	13.6%	44.4%
Fibroblasts**	142	51	25	17.6%	49.0%
Hofbauer Cells	242	343	160	66.1%	46.6%
Leukocytes***	1031	53	51	4.9%	96.2%
Syncytiotrophoblast	13	233	1	7.7%	0.4%
Mean:	261	105	37	17.5%	39.2%

*Single-cell subtypes cytotrophoblasts and proliferative cytotrophoblasts collapsed to single category
**Single-cell subtypes fibroblasts and mesenchymal stem cells collapsed to single category
***Single-cell peripheral immune cell subtypes collapsed to single category

Supplementary Table 3. Number of overlapping differentially upregulated genes and overrepresented biological process pathways between the single- and sorted cell type differential expression and enrichment testing analyses.

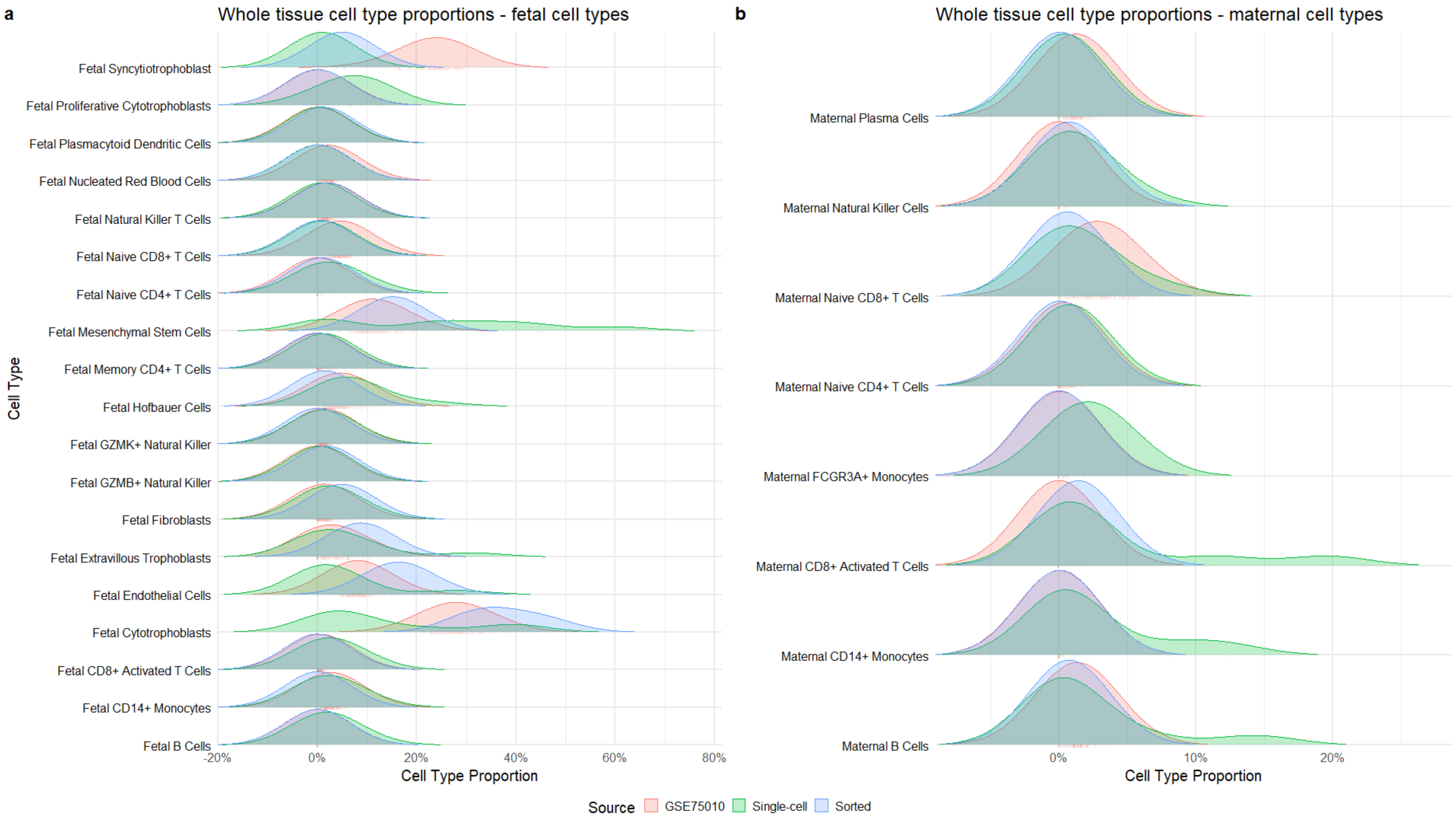

Supplementary Figure 13. Distribution of estimated cell type proportions in whole placental villous tissue from Michigan samples and GSE75010 controls compared to the number of single cells captured in the single-cell RNA sequencing datasets. Density distribution is colored by study source. (a) Fetal cell types. (b) Maternal cell types.

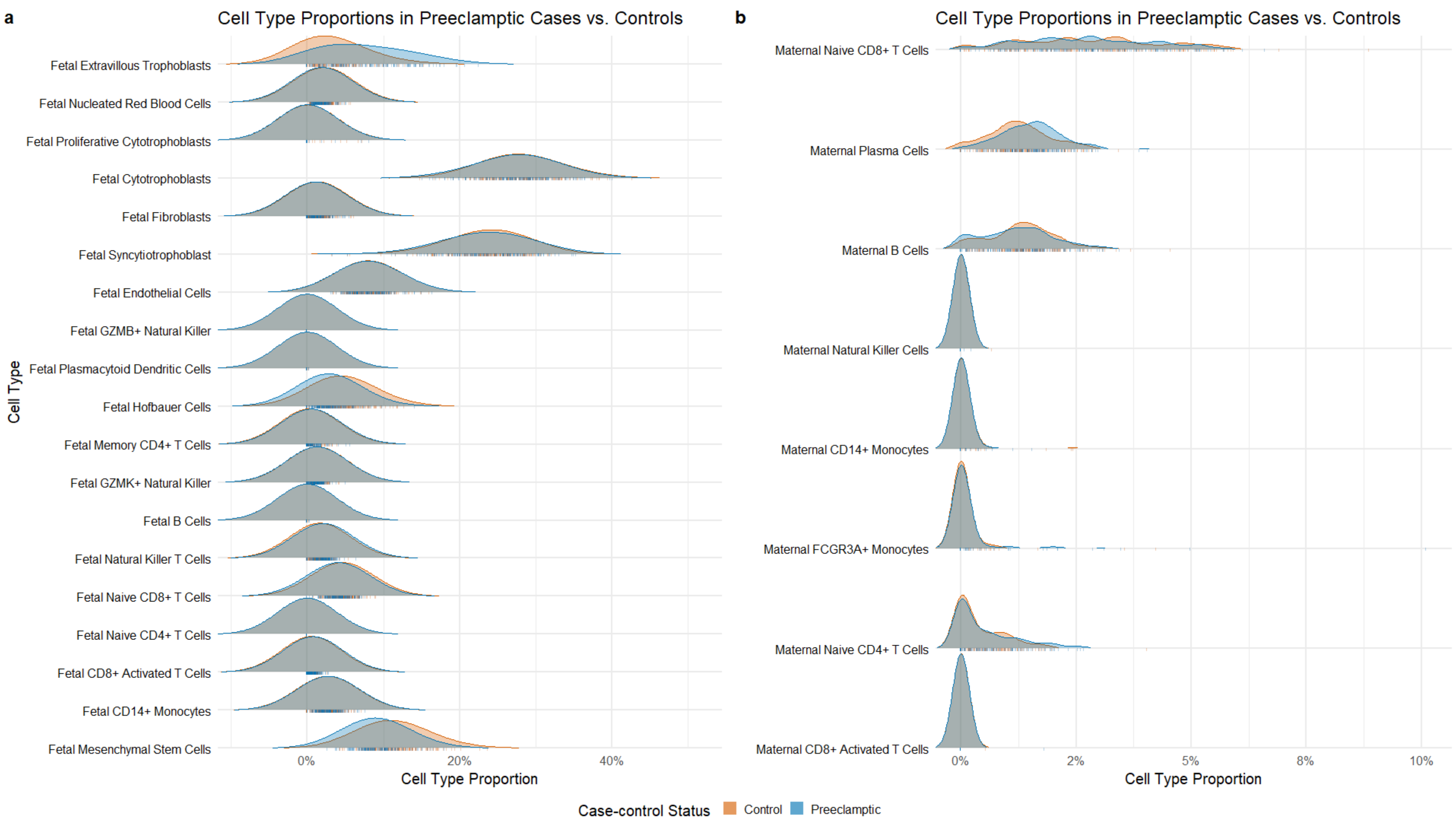

Supplementary Figure 14. Distribution of estimated cell type proportions in preeclamptic cases versus controls. Density distribution is colored by case-control status. (a) Fetal cell types. (b) Maternal cell types.

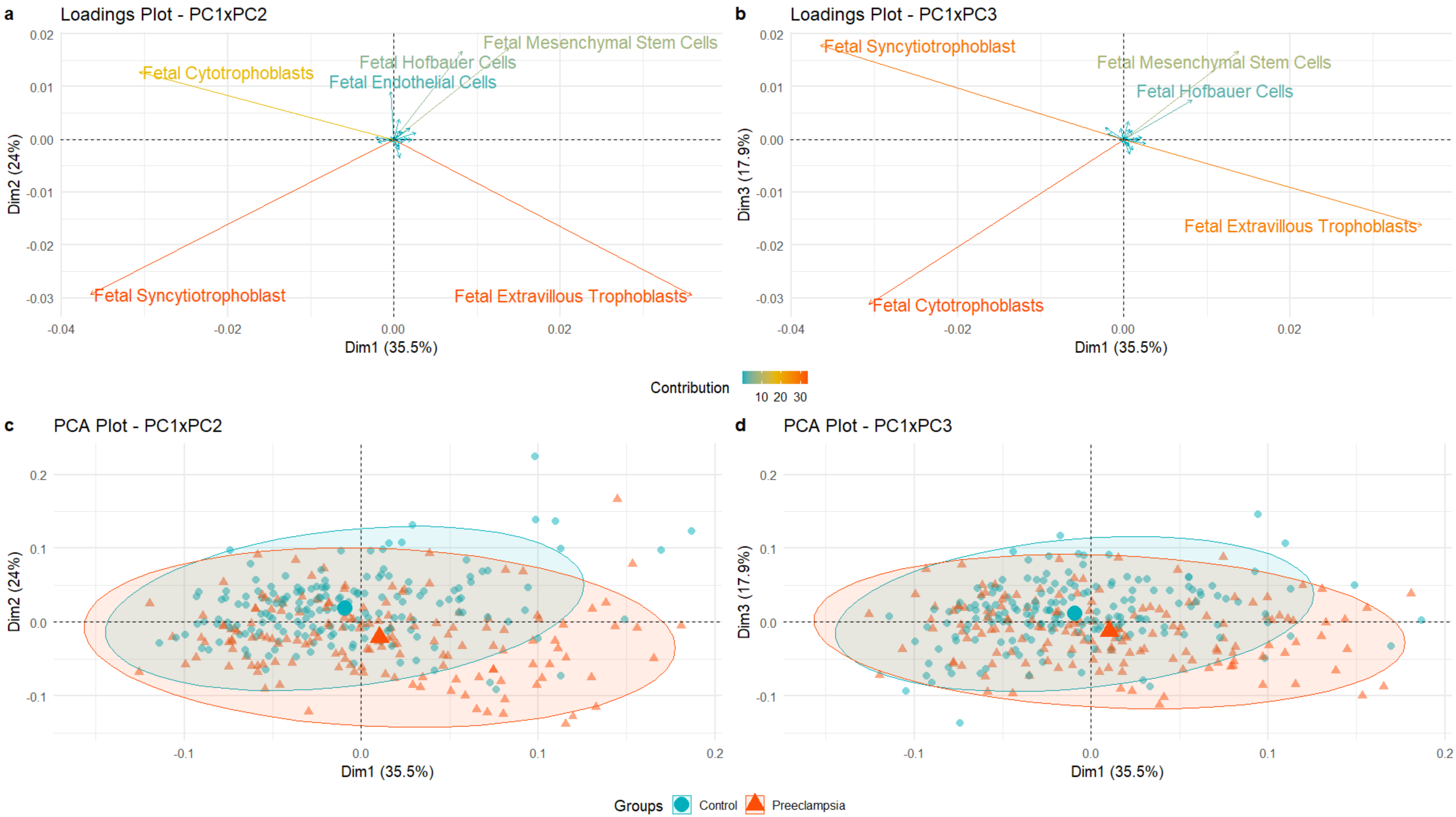

Supplementary Figure 15. Principal component (PC) results of estimated cell type proportions. Contribution refers to the relative proportions (expressed as a percentage) of the variation in a principal component attributable to an individual cell type. (a) PC1 and PC2 dimension loadings are largely driven by fetal syncytiotrophoblasts and fetal extravillous trophoblasts. (b) PC1 and PC3 loadings are largely driven by fetal syncytiotrophoblasts, fetal extravillous trophoblasts, and fetal cytotrophoblasts. (c) Individual observations projected onto PC1xPC2, with observations colored and shape-coded by preeclampsia case-control status. (d) Individual observations projected onto PC1xPC3, with observations colored and shape-coded by preeclampsia case-control status.

Supplementary References

1. Pique-Regi R, Romero R, Tarca AL, et al (2019) Single cell transcriptional signatures of the human placenta in term and preterm parturition. eLife 8:. https://doi.org/10.7554/eLife.52004

2. Tsang JCH, Vong JSL, Ji L, et al (2017) Integrative single-cell and cell-free plasma RNA transcriptomics elucidates placental cellular dynamics. PNAS 114:E7786–E7795. https://doi.org/10.1073/pnas.1710470114
