# Supplementary material for: Placental cell type deconvolution reveals that cell proportions drive preeclampsia gene expression differences
Source: Commun Biol. 2023 Mar 13;6:264. doi: 10.1038/s42003-023-04623-6 (PMC10011423; doi:10.1038/s42003-023-04623-6)
Supplement: Supplementary file 3 — Description of Additional Supplementary Data [file 42003_2023_4623_MOESM3_ESM.docx]

**Description of Additional Supplementary Files**

**File name:** Supplementary Data 1

**Description:** Differential expression results comparing proliferative vs. non-proliferative cytotrophoblasts using Seurat’s FindMarkers function. Differential expression sheet columns describe cluster cell type identity, marker gene symbol, average log fold change between this cell type cluster and other cell type clusters, prevalence of marker gene expression in cell type cluster, prevalence of marker gene expression in other cell type clusters, nominal p-value, and Bonferroni-adjusted p-value. Table columns describe gene ontology term name, false discovery rate-controlled q-value, the size of the gene ontology term, the number of top ranked genes used to test for enrichment against that term, the ontology source, and the ontology source ID.

**File name:** Supplementary Data 2

**Description:** Top marker gene expression (Bonferroni-adjusted p-value < 0.05) with highest average log fold change for each cell type cluster using Seurat’s FindAllMarkers function. Table columns describe cluster cell type identity, marker gene symbol, average log fold change between this cell type cluster and other cell type clusters, prevalence of marker gene expression in cell type cluster, prevalence of marker gene expression in other cell type clusters, nominal p-value, and Bonferroni-adjusted p-value.

**File name:** Supplementary Data 3

**Description:** Results from g:Profiler2’s g:GOSt functional enrichment function using cell type overexpressed genes from Supplementary Data 2 against the Gene Ontology Biological Process database. Table columns describe cell type cluster, gene ontology biological process term name, adjusted p-value, the size of the gene ontology term, and the number of top ranked genes used to test for enrichment against that term.

**File name:** Supplementary Data 4

**Description:** Differentially upregulated genes sorted by descending test statistic from DESeq2 differential expression analysis comparing expression one placental cell type against average expression in other placental cell types, adjusted for sample source. Cell type describes the cell type of interest for the contrast. Gene refers to the name of the genomic feature testing. Log2 Fold-change is log2-transformed effect size of the tested gene. Base mean is the average normalized count values. P-value is the nominal p-value. q-value is the false discovery-controlled cutoff at 0.05

**File name:** Supplementary Data 5

**Description:** Results from g:Profiler2’s g:GOSt functional enrichment function using cell type overexpressed genes from Supplementary Data 4 against the Gene Ontology Biological Process database. Table columns describe cell type cluster, gene ontology biological process term name, adjusted p-value, the size of the gene ontology term, and the number of top ranked genes used to test for enrichment against that term.

**File name:** Supplementary Data 6

**Description:** Estimated cell type proportions for the 4 whole tissue (with 1 additional technical replicate) and 19 sorted cell type fractions from the bulk RNA-sequencing results. On sheet 1, sample corresponds to the individual sample; cell type describes the target cell type fraction; and each subsequent column describes the estimated proportion for each cell type. On sheet 2, sample corresponds to the individual sample; each cell type is listed; p-value corresponds to the goodness-of-fit test for deconvolution (a statistically significant result suggests the signature gene reference matrix and the bulk mixture came from the same tissue); correlation describes the correlation between the original mixture and the estimated mixture among the signature genes; and RMSE is the root mean-square error between the original mixture and the imputed mixture among signature genes.

**File name:** Supplementary Data 7

**Description:** Estimated cell type proportions for preeclampsia bulk tissue dataset (GSE75010). Mixture corresponds to the individual microarray observations; each cell type is listed; p-value corresponds to the goodness-of-fit test for deconvolution (a statistically significant result suggests the signature gene reference matrix and the bulk mixture came from the same tissue); correlation describes the correlation between the original mixture and the estimated mixture among the signature genes; and RMSE is the root mean-square error between the original mixture and the imputed mixture among signature genes.

**File name:** Supplementary Data 8

**Description:** Full beta regression model results for the preeclamp

analysis. Each cell type proportion outcome is presented on a different Excel sheet. The preeclampsia parameter estimates for each cell type are presented in Figure 3. Component denotes whether the regression term corresponds to mean estimates for the parameters of interest or the precision term for the model; term corresponds to the name of the independent variable included in the model; estimate corresponds to the mean effect estimate of the model on the logit scale; std.error corresponds to the standard error of the mean estimate on the logit scale; statistic corresponds to the associated test statistic; p.value corresponds to the p.value for the associated test statistic; conf.low represents the lower bound of the 95% confidence interval on the logit scale; conf.high corresponds to the upper bound of the 95% confidence interval on the logit scale; or corresponds to the prevalence odds ratio estimate; conf.low.or represents the lower bound of the prevalence odds ratio 95% confidence interval; and conf.high.or represents the upper bound of the prevalence odds ratio 95% confidence interval.

**File name:** Supplementary Data 9

**Description:** Full differential expression results for the preeclampsia case-control differential expression analysis presented in Figure 4. The results for the base model and the cell type adjusted model are presented on separate Excel sheets. Log2 Fold Change corresponds to the log2 fold change for the expression of that gene in preeclampsia cases compared to controls; average expression corresponds the average expression of that gene across all arrays and channels; moderated t-statistic corresponds to the empirical Bayes standard error moderated t-statistic for differential expression.

**File name:** Supplementary Data 10

**Description:** Full differential expression enrichment results for the preeclampsia case-control differential expression analysis presented in Figure 5. The results for the base model and the cell type adjusted model are presented on separate Excel sheets.
