# Supplementary material for: Placental cell type deconvolution reveals that cell proportions drive preeclampsia gene expression differences
Source: Commun Biol. 2023 Mar 13;6:264. doi: 10.1038/s42003-023-04623-6 (PMC10011423; doi:10.1038/s42003-023-04623-6)
Supplement: Supplementary file 14 — Reporting Summary Checklist [file 42003_2023_4623_MOESM14_ESM.pdf]

nature portfolio

Corresponding author(s): Kelly Bakulski, PhD

Last updated by author(s): Feb 9, 2023

Reporting Summary

Nature Portfolio wishes to improve the reproducibility of the work that we publish. This form provides structure for consistency and transparency in reporting. For further information on Nature Portfolio policies, see our Editorial Policies and the Editorial Policy Checklist.

Statistics

For all statistical analyses, confirm that the following items are present in the figure legend, table legend, main text, or Methods section.

n/a	Confirmed	
☐	☒	The exact sample size (*n*) for each experimental group/condition, given as a discrete number and unit of measurement
☐	☒	A statement on whether measurements were taken from distinct samples or whether the same sample was measured repeatedly
☐	☒	The statistical test(s) used AND whether they are one- or two-sided<br>*Only common tests should be described solely by name; describe more complex techniques in the Methods section.*
☐	☒	A description of all covariates tested
☐	☒	A description of any assumptions or corrections, such as tests of normality and adjustment for multiple comparisons
☐	☒	A full description of the statistical parameters including central tendency (e.g. means) or other basic estimates (e.g. regression coefficient) AND variation (e.g. standard deviation) or associated estimates of uncertainty (e.g. confidence intervals)
☐	☒	For null hypothesis testing, the test statistic (e.g. *F*, *t*, *r*) with confidence intervals, effect sizes, degrees of freedom and *P* value noted<br>*Give P values as exact values whenever suitable.*
☒	☐	For Bayesian analysis, information on the choice of priors and Markov chain Monte Carlo settings
☒	☐	For hierarchical and complex designs, identification of the appropriate level for tests and full reporting of outcomes
☐	☒	Estimates of effect sizes (e.g. Cohen's *d*, Pearson's *r*), indicating how they were calculated

Our web collection on statistics for biologists contains articles on many of the points above.

Software and code

Policy information about availability of computer code

Data collection	No software was used for data collection.
Data analysis	All scripts to perform preprocessing and analyses is available (https://zenodo.org/badge/latestdoi/599836831). Detailed software information, including versions can be found in the methods.

For manuscripts utilizing custom algorithms or software that are central to the research but not yet described in published literature, software must be made available to editors and reviewers. We strongly encourage code deposition in a community repository (e.g. GitHub). See the Nature Portfolio guidelines for submitting code & software for further information.

Data

Policy information about availability of data

All manuscripts must include a data availability statement. This statement should provide the following information, where applicable:

- Accession codes, unique identifiers, or web links for publicly available datasets
- A description of any restrictions on data availability
- For clinical datasets or third party data, please ensure that the statement adheres to our policy

Data citations are provided in the manuscript. Raw placental single-cell RNA-sequencing and raw placental bulk RNA-sequencing generated by this study are freely available in the Gene Expression Omnibus repository (accession number GSE182381). The cell type signature matrix and related files to deconvolute bulk gene expression measures are available through the Gene Expression Omnibus (accession number GSE182381) as supplementary material for download. This study makes use of data generated by The Chinese University of Hong Kong (CUHK) Circulating Nucleic Acids Research Group, as reported by Tsang et al in Proc Natl Acad Sci USA (doi: 10.1073/pnas.1710470114, accession number EGAS00001002449). The placental single-cell RNA-sequencing data that support the findings of this study can be accessed through the Database of Genotypes and Phenotypes (accession number phs001886.v1.p1) and the European Genome-Phenome Archive

(accession number EGAS00001002449). The preeclampsia case-control microarray data that support the findings of this study are available in Gene Expression Omnibus repository (accession number GSE75010).

Field-specific reporting

Please select the one below that is the best fit for your research. If you are not sure, read the appropriate sections before making your selection.

☐ Life sciences ☒ Behavioural & social sciences ☐ Ecological, evolutionary & environmental sciences

For a reference copy of the document with all sections, see nature.com/documents/nr-reporting-summary-flat.pdf

Behavioural & social sciences study design

All studies must disclose on these points even when the disclosure is negative.

Study description	The placenta mediates adverse pregnancy outcomes, including preeclampsia, characterized by gestational hypertension and proteinuria. Placental cell type heterogeneity in preeclampsia is not well-understood and limits mechanistic interpretation of bulk gene expression measures. We generated single-cell RNA-sequencing samples for integration with existing data to create the largest deconvolution reference of 19 fetal and 8 maternal cell types from placental villous tissue at term. We deconvoluted eight published microarray case-control studies of preeclampsia. Our findings indicate substantial placental cellular heterogeneity in preeclampsia that predict previously observed bulk gene expression differences. Our deconvolution reference lays the groundwork for cellular heterogeneity-aware investigation into placental dysfunction and adverse birth outcomes.
Research sample	Clinically excess placental tissues were collected in a convenience sample shortly after delivery from healthy, full term, singleton uncomplicated Cesarean sections at the University of Michigan Von Voigtlander Women's Hospital. This is an appropriate sample to answer this study question because cell type phenotype is conserved across individuals. Selected samples from dbGaP ID phs001886.v1.p1 were generated on an identical platform in the same tissue in a similar population and so were included. Selected samples from European Genome-Phenome Archive (accession number EGAS00001002449) were generated on an earlier version of the same platform in the same tissue in a similar population and so were also included. This is not a representative population.
Sampling strategy	This study employed a convenience sampling strategy. Sample sizes were predetermined to meet the minimum threshold for statistical testing via methods employed in this study.
Data collection	Data collection was limited to bulk or single-cell RNA-sequencing. The University of Michigan Advanced Genomics Core was blind to the experimental condition and study hypothesis during sequencing and its preparation.
Timing	Primary samples were collected during July-September, 2017.
Data exclusions	In the single-cell RNA-sequencing experiment, doublets were removed as well as cells that did not meet quality control criteria as described in the Methods. Deconvoluted cell type estimates in dataset GSE75010 were excluded from downstream analyses if their estimated abundance was 0% across all samples.
Non-participation	This study does not meet the definition of human subjects research; therefore, non-participatation was not relevant.
Randomization	Batch effects were controlled for using assay-appropriate techniques and/or other covariates were included in regression models where appropriate.

Reporting for specific materials, systems and methods

We require information from authors about some types of materials, experimental systems and methods used in many studies. Here, indicate whether each material, system or method listed is relevant to your study. If you are not sure if a list item applies to your research, read the appropriate section before selecting a response.

Materials & experimental systems

n/a	Involved in the study	
☐	☒	Antibodies
☒	☐	Eukaryotic cell lines
☒	☐	Palaeontology and archaeology
☒	☐	Animals and other organisms
☒	☐	Human research participants
☒	☐	Clinical data
☒	☐	Dual use research of concern

Methods

n/a	Involved in the study	
☒	☐	ChIP-seq
☐	☒	Flow cytometry
☒	☐	MRI-based neuroimaging

Antibodies

Antibodies used	FITC, marker CD9: Mouse IgG1-kappa, clone HI9a (2.5 µg/mL), Biolegend #312103, lot B188319, Biolegend #312104, lot B232916;

Antibodies used	isotype control: clone MOPC-21 Biolegend #400107, Lot B199152 (2.5 µg/mL). APC, marker CD45: Mouse IgG1-kappa, clone 2D1, Biolegend #368511, Lot B215062 (0.125 µg/mL); isotype control: clone MOPC-21, Biolegend #400121, lot B216780 (0.125 µg/mL). PE/CY-7, marker HLA-ABC: Mouse IgG2a-kappa, clone W6/32, Biolegend #311429, lot B188649, Biolegend #3111430, lot B238602 (0.44 µg/mL); isotype control: clone MOPC-173, Biolegend #400231, lot B209000 (0.44 µg/mL);. BV421, marker CD31: Mouse IgG1-kappa, clone WM59, Biolegend #303123, lot B204347, Biolegend #303124, lot B232010 (0.625 µg/mL); isotype control: clone MOPC-21, Biolegend #400157, lot B225357 (0.625 µg/mL). PE, marker HLA-G: Mouse IgG2a-kappa, clone 87G, Biolegend #335905, lot B222326, Biolegend #335906, lot B199294 (5 µg/mL); isotype control clone MOPC-173, Biolegend #400211, lot B227641 (5 µg/mL). Mouse IgG1-kappa, clone MEM-G/9, Abcam #24384 Lot GR3176304-1 (2.5 µg/mL); isotype control: monoclonal, Abcam #ab81200, lot GR267131-1 (2.5 µg/mL).
Validation	Validation information available on manufacturer's website under the catalog ID for each antibody.

Flow Cytometry

Plots

Confirm that:

☒ The axis labels state the marker and fluorochrome used (e.g. CD4-FITC).

☒ The axis scales are clearly visible. Include numbers along axes only for bottom left plot of group (a 'group' is an analysis of identical markers).

☒ All plots are contour plots with outliers or pseudocolor plots.

☒ A numerical value for number of cells or percentage (with statistics) is provided.

Methodology

Sample preparation	Placentas were collected shortly after delivery from healthy, full term, singleton uncomplicated Cesarean sections at the University of Michigan Von Voigtlander Women's Hospital. Villous placental tissue biopsies were collected and minced for dissociation after cutting away the basal and chorionic plates and scraping villous tissue from blood vessels. We subjected approximately 1g minced dissected villous tissue to the Miltenyi Tumor Dissociation Kit on the GentleMACS Octo Dissociator with Heaters (Miltenyi Biotec) to yield single-cell suspensions of viable placental cells in 5µM StemMACS™ Y27632 (Miltenyi Biotec) in RPMI 1640 (Gibco) according to manufacturer's instructions for "soft" tumor type. Red blood cells were depleted using RBC lysis buffer (Biolegend) according to manufacturer's protocol A. Single-cell suspensions were size-filtered at 100µm and subsequently 40µm. Single-cell suspensions <40µm were cryogenically stored in 5µM StemMACS™ Y27632 90% heat-inactivated fetal bovine serum (Gibco)/10% dimethyl sulfoxide (Invitrogen). Villous tissue single-cell suspensions were quickly thawed and stained with 5 fluorescently labeled antibodies (CD9-FITC, CD45-APC, HLA-I-PE/Cy7, CD31-BV421, and HLA-G-PE) as well as the LIVE/DEAD Near-IR stain (Invitrogen) to isolate 6 viable populations of placental cells by fluorescence activated cell sorting at the University of Michigan Flow Cytometry Core Facility.
Instrument	Beckman Coulter MoFlo Astrios
Software	DeNovo Software's FCS Express
Cell population abundance	Post-sorting abundance data available in Supplementary Table 4. Purity was assessed based on complete staining profiles and bulk RNA-sequencing results.
Gating strategy	A cut-off of 0.1% events was used to set a series of gates. Cells were first gated on size and granularity (FSC-HxSSC-H) to eliminate debris, followed by doublet discrimination (FSC-HxFSC-W and SSC-HxSSC-W). Ax750 was used to sort on viability. Extravillous trophoblasts were isolated based on Human Leukocyte Antigen-G (HLA-G) expression (Supplementary Figure 10a). Cytotrophoblasts are HLA-ABC negative (Supplementary Figure 10b). HLA-ABC positive cells were then subjected to a CD45/CD9 gate to isolate Hofbauer cells and a heterogeneous population of leukocytes (Supplementary Figure 10c). Finally, CD45-/CD9- population is sorted into the endothelial or fibroblast bins based on CD31 expression (Supplementary Figure 10d). Initial flow cytometry experiments included fluorescence minus one, single color compensation, and isotype controls. Isotype controls were found to be the most conservative and were consequently included in all sorting experiments, as well as single-color compensation controls due to the large number of colors used in sorting.

☒ Tick this box to confirm that a figure exemplifying the gating strategy is provided in the Supplementary Information.
